# A Comprehensive Review of Chloride Management in Critically Ill Patients

**DOI:** 10.7759/cureus.55625

**Published:** 2024-03-06

**Authors:** Nandhini Sagar, Sham Lohiya

**Affiliations:** 1 Pediatrics, Jawaharlal Nehru Medical College, Datta Meghe Institute of Higher Education and Research, Wardha, IND

**Keywords:** balanced solutions, acid-base balance, fluid resuscitation, electrolyte imbalances, critically ill patients, chloride management

## Abstract

Chloride, often overshadowed in electrolyte management, emerges as a crucial player in the physiological intricacies of critically ill patients. This comprehensive review explores the multifaceted aspects of chloride, ranging from its significance in cellular homeostasis to the consequences of dysregulation in critically ill patients. The pathophysiology of hyperchloremia and hypochloremia is dissected, highlighting their intricate impact on acid-base balance, renal function, and cardiovascular stability. Clinical assessment strategies, including laboratory measurements and integration with other electrolytes, lay the foundation for targeted interventions. Consequences of dysregulated chloride levels underscore the need for meticulous management, leading to an exploration of emerging therapies and interventions. Fluid resuscitation protocols, the choice between crystalloids and colloids, the role of balanced solutions, and individualized patient approaches comprise the core strategies in chloride management. Practical considerations, such as monitoring and surveillance, overcoming implementation challenges, and embracing a multidisciplinary approach, are pivotal in translating theoretical knowledge into effective clinical practice. As we envision the future, potential impacts on critical care guidelines prompt reflections on integrating novel therapies, individualized approaches, and continuous monitoring practices. In conclusion, this review synthesizes current knowledge, addresses practical considerations, and envisions future directions in chloride management for critically ill patients. By embracing a holistic understanding, clinicians can navigate the complexities of chloride balance, optimize patient outcomes, and contribute to the evolving landscape of critical care medicine.

## Introduction and background

Chloride is an essential anion. In adults, the normal range for chloride is 96-106 milliequivalents per liter (mEq/L). It is pivotal in maintaining electrolyte balance within the human body. Its significance extends beyond a mere salt constituent; chloride actively participates in various physiological processes crucial for cellular function [1]. Chloride, denoted as Cl^-, is a negatively charged ion that pairs with sodium to form sodium chloride (NaCl), commonly known as table salt. While sodium often receives more attention, chloride is equally indispensable. It is a crucial electrolyte, maintaining osmotic pressure, acid-base balance, and electrical neutrality in bodily fluids [2]. Beyond its electrochemical role, chloride is intricately involved in cellular homeostasis, influencing cell membrane potential and aiding in transporting nutrients and waste products. Its significance spans multiple organ systems, with notable impacts on the kidneys, cardiovascular system, and neuromuscular function [3].

In critically ill patients, maintaining electrolyte balance takes on heightened significance. Dysregulated chloride levels have been associated with adverse outcomes, including metabolic acidosis, impaired organ function, and disruptions in hemodynamic stability. The intricate interplay between chloride and other electrolytes necessitates a nuanced approach to its management, particularly in the dynamic and often challenging critical care environment [4]. Understanding the specific challenges that critically ill patients face, such as fluid resuscitation, altered renal function, and the potential for rapid electrolyte shifts, underscores the importance of a targeted focus on chloride management. This rationale forms the basis for exploring strategies that optimize chloride levels in critical care, improving patient outcomes and reducing complications associated with electrolyte imbalances [5]. This review aims to provide a comprehensive examination of chloride management in critically ill patients. By synthesizing current knowledge, exploring the physiological intricacies of chloride regulation, and evaluating evidence-based practices, this review aims to offer clinicians, researchers, and healthcare providers a thorough understanding of chloride's complexities in critical care settings.

## Review

Pathophysiology of chloride imbalance in critical illness

The causes of hyperchloremia and hypochloremia are mentioned in Table 1 [6-10].

**Table 1:** Causes of hyperchloremia and hypochloremia

Causes of hyperchloremia	Causes of hypochloremia
Excessive saline administration: The administration of large volumes of normal saline, a chloride-rich solution, is a common practice in various clinical settings, especially during resuscitation or fluid replacement. Normal saline, comprising 0.9% sodium chloride, can contribute to hyperchloremia when administered in excess. Elevated chloride levels can disrupt the balance of electrolytes in the blood, leading to a state known as hyperchloremia. This phenomenon is often associated with non-anion gap metabolic acidosis. In this context, the increase in chloride is not balanced by a corresponding increase in anions, leading to a relative excess of chloride ions. The resulting acidosis is characterized by decreased serum bicarbonate levels, impacting the overall acid-base balance [6].	Fluid loss: Hypochloremia, a state characterized by lower-than-normal levels of chloride in the blood, can arise from excessive loss of chloride through various mechanisms. Gastrointestinal fluids, including those lost through vomiting or diarrhea, represent a significant route for chloride depletion. In such cases, the body may lose chloride-rich fluids, disrupting the balance of electrolytes in the blood. Additionally, renal mechanisms can contribute to hypochloremia when the kidneys excrete excess chloride, reducing circulating levels. Clinical conditions such as Addison's disease, which affects the adrenal glands, or the use of diuretics that enhance renal chloride excretion may exacerbate chloride depletion and contribute to the development of hypochloremia [9].
Impaired renal function: The kidneys play a crucial role in maintaining electrolyte balance, including the reabsorption and excretion of chloride. Dysfunction in the tubular reabsorption process can occur due to various renal conditions, leading to impaired chloride excretion. In such cases, the kidneys may struggle to regulate chloride levels effectively, contributing to elevated serum chloride concentrations. Impaired renal function, whether acute or chronic, can thus be a significant factor in developing hyperchloremia. This underscores the intricate relationship between renal health and chloride homeostasis in the body [7].	Metabolic alkalosis: Hypochloremia is often associated with metabolic alkalosis, characterized by elevated blood pH and bicarbonate levels. This association is particularly notable in prolonged vomiting or excessive bicarbonate administration. During vomiting, the loss of hydrochloric acid (which contains chloride ions) contributes to reducing circulating chloride levels. Additionally, if there is an excess administration of bicarbonate or other alkaline substances, the relative decrease in chloride concentration can contribute to the alkalotic state. The interplay between chloride levels and acid-base balance underscores the significance of hypochloremia in contributing to metabolic alkalosis [10].
Acid-base disorders: Hyperchloremia is closely associated with metabolic acidosis, a condition characterized by decreased blood pH and bicarbonate levels. The connection between hyperchloremia and acidosis becomes particularly evident with an increased chloride-to-sodium ratio. In normal physiological conditions, the balance between chloride and sodium is maintained to preserve electroneutrality. However, in scenarios such as renal failure or diabetic ketoacidosis, this balance may be disrupted. When not accompanied by a proportional increase in sodium, the elevated chloride levels contribute to metabolic acidosis. Understanding the link between hyperchloremia and acid-base disorders is crucial for clinicians in diagnosing and managing the underlying conditions that may lead to this electrolyte imbalance [8].	Syndromes and disorders: Certain syndromes and disorders can lead to hypochloremia due to abnormalities in chloride transport mechanisms in the kidneys. Bartter syndrome, both inherited and acquired forms, is a notable example. This syndrome is characterized by renal tubular defects affecting electrolyte reabsorption, including chloride. As a result, individuals with Bartter syndrome may experience chronic hypochloremia. Understanding the syndromes and disorders that impact chloride transport provides valuable insights into the underlying mechanisms of hypochloremia and guides targeted approaches to management and treatment [11].

Causes of Hyperchloremia

Excessive saline administration: The administration of large volumes of normal saline, a chloride-rich solution, is a common practice in various clinical settings, especially during resuscitation or fluid replacement. Normal saline, comprising 0.9% sodium chloride, can contribute to hyperchloremia when administered in excess. The elevated chloride levels can disrupt the balance of electrolytes in the blood, leading to a state known as hyperchloremia. This phenomenon is often associated with non-anion gap metabolic acidosis. In this context, the increase in chloride is not balanced by a corresponding increase in anions, leading to a relative excess of chloride ions. The resulting acidosis is characterized by decreased serum bicarbonate levels, impacting the overall acid-base balance [6].

Impaired renal function: The kidneys play a crucial role in maintaining electrolyte balance, including the reabsorption and excretion of chloride. Dysfunction in the tubular reabsorption process can occur due to various renal conditions, such as the extrarenal generation of metabolic alkalosis, which leads to the excretion of bicarbonate into the urine, which obligates a component of filtered sodium to accompany the base, leading to impaired chloride excretion. In such cases, the kidneys may struggle to regulate chloride levels effectively, contributing to elevated serum chloride concentrations. Impaired renal function, whether acute or chronic, can thus be a significant factor in developing hyperchloremia. Malfunctions in these channels can lead to impaired absorption of ions and ultimately alter the osmotic balance in the kidney, with consequences on the ionic balance of the blood and tissues of the body. This underscores the intricate relationship between renal health and chloride homeostasis in the body [7].

Acid-base disorders: Hyperchloremia is closely associated with metabolic acidosis, a condition characterized by decreased blood pH and bicarbonate levels. The connection between hyperchloremia and acidosis becomes particularly evident with an increased chloride-to-sodium ratio. In normal physiological conditions, the balance between chloride and sodium is maintained to preserve electroneutrality. However, in scenarios such as renal failure or diabetic ketoacidosis, this balance may be disrupted. When not accompanied by a proportional increase in sodium, the elevated chloride levels contribute to metabolic acidosis. Understanding the link between hyperchloremia and acid-base disorders is crucial for clinicians in diagnosing and managing the underlying conditions that may lead to this electrolyte imbalance [8].

Causes of Hypochloremia

Fluid loss: Hypochloremia, a state characterized by lower-than-normal levels of chloride in the blood, can arise from excessive loss of chloride through various mechanisms. Gastrointestinal fluids, including those lost through vomiting or diarrhea, represent a significant route for chloride depletion. In such cases, the body may lose chloride-rich fluids, disrupting the balance of electrolytes in the blood. Additionally, renal mechanisms can contribute to hypochloremia when the kidneys excrete excess chloride, reducing circulating levels. Clinical conditions such as Addison's disease, which affects the adrenal glands, or the use of diuretics that enhance renal chloride excretion may exacerbate chloride depletion and contribute to the development of hypochloremia [9].

Metabolic alkalosis: Hypochloremia is often associated with metabolic alkalosis, characterized by elevated blood pH and bicarbonate levels. Hydrogen ions are derived from the dissociation of water into hydrogen and hydroxyl ions; therefore, when hydrogen ions are removed from the extracellular fluid, the remaining hydroxyl ion combines with carbon dioxide to form bicarbonate. Gastrointestinal and renal hydrogen loss is usually accompanied by the loss of chloride and potassium, resulting in hypochloremia and hypokalemia. Patients with preserved kidney function should be able to rapidly excrete excess bicarbonate in the urine. Thus, metabolic alkalosis can only persist if the ability to excrete excess bicarbonate in the urine is impaired due to one of the following causes: hypovolemia, reduced effective arterial blood volume (due, for example, to heart failure or cirrhosis), chloride depletion, hypokalemia, reduced glomerular filtration rate, hyperaldosteronism or similar disorders, or combinations of these factors. This association is particularly notable in prolonged vomiting or excessive bicarbonate administration. During vomiting, the loss of hydrochloric acid (which contains chloride ions) contributes to reducing circulating chloride levels. Additionally, if there is an excess administration of bicarbonate or other alkaline substances, the relative decrease in chloride concentration can contribute to the alkalotic state. The interplay between chloride levels and acid-base balance underscores the significance of hypochloremia in contributing to metabolic alkalosis [10].

Syndromes and disorders: Certain syndromes and disorders can lead to hypochloremia due to abnormalities in chloride transport mechanisms in the kidneys. Bartter syndrome, both inherited and acquired forms, is a notable example. This syndrome is characterized by renal tubular defects affecting electrolyte reabsorption, including chloride. As a result, individuals with Bartter syndrome may experience chronic hypochloremia. Understanding the syndromes and disorders that impact chloride transport provides valuable insights into the underlying mechanisms of hypochloremia and guides targeted approaches to management and treatment [11].

Impact on Organ Function

Renal implications: Dysregulation of chloride levels significantly affects renal function, primarily influencing tubular reabsorption and secretion within the kidneys. The impaired regulation of chloride can contribute to alterations in glomerular filtration rates, disrupting the kidney's ability to filter and excrete substances effectively. The result may be the development of electrolyte imbalances, as the intricate balance of chloride, sodium, and other electrolytes is compromised. This underscores the critical role of chloride in maintaining renal homeostasis and emphasizes the potential impact of its dysregulation on overall kidney function [12].

Cardiovascular effects: Chloride levels are pivotal in cardiovascular dynamics, and imbalances can lead to significant cardiovascular consequences. Hyperchloremia, characterized by elevated levels of chloride, has been associated with adverse cardiovascular outcomes. This includes decreased cardiac output, myocardial contractility impairments, and microcirculation disturbances. These effects may contribute to hemodynamic instability and compromise tissue perfusion in critically ill patients. Conversely, hypochloremia, with lower chloride levels, can contribute to cardiovascular instability by predisposing individuals to arrhythmias. Severe cases may lead to cardiovascular collapse, highlighting chloride's delicate balance in cardiovascular health [13].

Neurological consequences: Electrolyte imbalances, including chloride levels, can profoundly affect neuronal excitability and function. Severe chloride imbalance, whether hyperchloremia or hypochloremia, may manifest in neurological symptoms. Hyperchloremia, with its associated metabolic acidosis, can impact the central nervous system and lead to symptoms such as confusion, lethargy, and, in extreme cases, seizures or coma. Hypochloremia, on the other hand, can also contribute to neurological manifestations, adding another layer of complexity to the consequences of chloride dysregulation. Recognizing and managing these neurological consequences are vital to effective chloride balance in the critically ill [14].

Clinical assessment of chloride status

Laboratory Measurement Techniques

Serum chloride measurement: The primary and most common method for assessing chloride levels involves blood tests that measure serum chloride concentrations. Serum chloride reflects the concentration of chloride ions in the extracellular fluid, providing a snapshot of the body's chloride status. This laboratory measurement is a fundamental tool in diagnosing and monitoring electrolyte imbalances, guiding clinicians in tailoring interventions to restore chloride equilibrium [15].

Blood gas analysis: Arterial blood gas (ABG) analysis is a critical component of assessing patient acid-base balance, encompassing the measurement of chloride levels. In critical care settings, ABG analysis offers real-time information about electrolyte status within the patient's overall acid-base equilibrium. Monitoring chloride levels alongside other blood gas parameters aids in diagnosing and managing acid-base disorders, providing valuable insights into the patient's physiological status [16].

Urinary chloride measurement: Assessing chloride levels in urine is a valuable diagnostic tool, particularly for understanding renal function and distinguishing between renal and non-renal causes of chloride imbalance. Measuring urinary chloride concentrations allows clinicians to evaluate the kidneys' chloride handling, providing information about tubular reabsorption and secretion. This insight is crucial in differentiating the underlying etiology of chloride disturbances and guiding appropriate therapeutic interventions [17].

Point-of-care testing: Advances in technology have led to the development of point-of-care testing devices designed to rapidly assess chloride levels at the patient's bedside. These devices offer immediate results, making them particularly valuable in emergencies or critical care settings where timely decision-making is essential. Point-of-care testing for chloride levels enhances the efficiency of clinical care, allowing for swift adjustments in treatment strategies based on real-time data [18].

Interpretation of Chloride Levels

The interpretation of chloride levels is mentioned in Table 2.

**Table 2:** Interpretation of serum chloride levels

Serum chloride levels	Value
Normal range	96-106 mmol/L
Hyperchloremia	>106 mmol/L
Hypochloremia	<96 mmol/L

Normal range: Understanding the normal reference range for serum chloride is fundamental for accurate clinical interpretation. The typical range for serum chloride levels is approximately 96-106 mmol/L. It is important to note that slight variations in normal ranges may exist depending on the specific laboratory conducting the analysis. Awareness of these variations ensures clinicians interpret results within the context of the laboratory's established reference values, facilitating precise assessments of chloride status in patients [19].

Hyperchloremia interpretation: Elevated serum chloride levels, known as hyperchloremia, may signify various underlying conditions. Excessive administration of normal saline, renal dysfunction impairing chloride excretion, or metabolic acidosis with an increased chloride-to-sodium ratio can contribute to this electrolyte imbalance. To interpret hyperchloremia accurately, clinicians must consider sodium levels and calculate the anion gap. This comprehensive assessment aids in identifying the specific cause, guiding targeted interventions, and addressing the broader clinical context contributing to elevated chloride levels [19].

Hypochloremia interpretation: Low serum chloride levels, or hypochloremia, can indicate diverse clinical conditions. Fluid loss through mechanisms such as vomiting or diarrhea, metabolic alkalosis, or syndromes such as Bartter syndrome may contribute to this electrolyte imbalance. Accurate interpretation of hypochloremia necessitates a thorough assessment involving other electrolytes and acid-base parameters. Understanding the interplay between chloride, sodium, bicarbonate, and other ions is critical for discerning the root cause of hypochloremia and tailoring interventions to address the underlying pathology [20].

Integration With Other Electrolytes

Sodium-chloride relationship: The symbiotic relationship between sodium and chloride maintains the body's osmotic pressure and fluid balance. Assessing chloride levels alongside sodium is essential, as alterations in their balance can provide valuable insights into specific clinical conditions. The chloride-to-sodium ratio is a crucial parameter, and an abnormal ratio may indicate underlying pathologies. An imbalance in this relationship can influence water distribution, cellular osmolarity, and overall fluid dynamics, emphasizing the interconnectedness of sodium and chloride in physiological homeostasis [14].

Electrolyte panel analysis: Integration with other electrolytes, such as potassium and bicarbonate, enhances the depth of understanding in assessing electrolyte balance. A comprehensive electrolyte panel analysis allows clinicians to evaluate the intricate interplay between different ions. Abnormalities in one electrolyte may impact the others, necessitating a holistic approach to clinical assessment. This broader perspective is particularly relevant in critically ill patients, where disturbances in electrolyte balance can have cascading effects on organ function and overall physiological stability [21].

Acid-base balance: Chloride's integral role in acid-base regulation makes its assessment pivotal in evaluating acid-base status. The anion gap, calculated as (sodium + potassium) - (chloride + bicarbonate), is valuable in diagnosing metabolic acidosis. This calculation reflects the unmeasured ions in the serum and helps identify the specific type of acidosis. An elevated anion gap may suggest lactic acidosis or ketoacidosis, guiding appropriate interventions. The nuanced relationship between chloride, bicarbonate, and other ions underscores the importance of considering acid-base balance in comprehensively evaluating chloride levels [22].

Consequences of dysregulated chloride levels

Acid-Base Balance Disruptions

Metabolic acidosis (hyperchloremic acidosis): Hyperchloremic acidosis is a metabolic acidosis characterized by elevated serum chloride levels contributing to reduced serum bicarbonate concentration. This disruption in acid-base balance can lead to a decrease in blood pH, affecting enzymatic function and cellular processes. The excess chloride ions, often associated with conditions such as excessive saline administration or renal dysfunction, disrupt the normal bicarbonate-chloride equilibrium. As a result, the body experiences an accumulation of chloride without a corresponding increase in bicarbonate, leading to an acidic environment. Hyperchloremic acidosis may have clinical implications for organ function, particularly affecting the kidneys and cardiovascular system [8].

Alkalosis (hypochloremic alkalosis): Hypochloremia can contribute to metabolic alkalosis, a condition characterized by an elevation in blood pH. This often occurs in situations where there is excessive loss of chloride-rich fluids, such as prolonged vomiting. The loss of hydrochloric acid (which contains chloride ions) in gastric secretions contributes to reduced chloride levels in the blood. The resultant electrolyte imbalance, with a relative decrease in chloride, can lead to metabolic alkalosis. This alkalotic state can manifest with symptoms of neuromuscular irritability and, in severe cases, may lead to seizures. Understanding the association between hypochloremia and metabolic alkalosis is crucial for diagnosing and managing these complex acid-base disturbances [23].

Anion gap changes: Dysregulated chloride levels directly impact the anion gap, a calculated parameter essential in diagnosing the underlying cause of metabolic acidosis. An increased anion gap, often observed in conditions such as lactic acidosis or ketoacidosis, reflects an excess of unmeasured anions in the blood. On the other hand, in hyperchloremic acidosis, the anion gap remains normal despite acidosis, as the excess chloride compensates for the reduction in bicarbonate. Recognizing changes in the anion gap helps clinicians narrow down the potential causes of metabolic acidosis and guides appropriate interventions based on the specific acid-base disorder [24].

Renal Implications

Tubular function disturbances: Dysregulation of chloride levels can profoundly affect tubular reabsorption and secretion in the kidneys, influencing overall renal function. Hyperchloremia, often associated with metabolic acidosis or excessive saline administration, has been linked to decreased glomerular filtration rates. This impairment in renal function can contribute to acute kidney injury (AKI), a severe concern in critical illness. The altered chloride balance can impact the delicate renal tubular processes, disrupting the intricate regulation of electrolytes and acid-base equilibrium within the kidneys [19].

Fluid and electrolyte balance: The kidneys play a central role in maintaining fluid and electrolyte balance in the body. Dysregulated chloride levels can disrupt this delicate equilibrium, leading to significant clinical consequences. Hyperchloremia may contribute to volume depletion or overload, affecting the overall fluid status of the patient. The imbalance in chloride levels can also influence the homeostasis of other electrolytes, such as sodium and potassium, with potential cascading effects on cellular function and overall renal perfusion. Understanding the impact of chloride on fluid and electrolyte balance is crucial for managing critically ill patients, particularly in resuscitation and fluid administration scenarios [4].

Nephrotoxicity: Prolonged hyperchloremia may contribute to nephrotoxicity, mainly when associated with certain medications or therapeutic interventions. Nephrotoxic effects may be accentuated in the context of critical illness, where the kidneys are already under stress. Monitoring and managing chloride levels become imperative in mitigating the risk of nephrotoxicity. Medications that contribute to hyperchloremia, such as those containing high chloride concentrations, may need to be carefully reconsidered in the overall treatment strategy to prevent potential harm to renal function. This highlights the importance of a nuanced approach to chloride management, considering the broader implications for renal health in critically ill patients [25].

Cardiovascular Effects

Impact on cardiac output: Hyperchloremia has been associated with decreased cardiac output and impaired myocardial contractility. The elevated levels of chloride, often seen in conditions such as metabolic acidosis or excessive saline administration, can negatively influence the cardiovascular system. The decrease in cardiac output and impaired myocardial function contribute to hemodynamic instability, compromising tissue perfusion in critically ill patients. Understanding the impact of hyperchloremia on cardiac function is crucial for managing the cardiovascular stability of patients in critical care settings [26].

Arrhythmias and cardiovascular collapse: Hypochloremia, mainly associated with metabolic alkalosis, can predispose patients to arrhythmias, such as ventricular tachycardia or fibrillation. The relative decrease in chloride levels, often due to conditions such as prolonged vomiting, contributes to the alkalotic state. This alteration in electrolyte balance can affect the electrical conductivity of the heart, leading to abnormal rhythms. In severe cases, hypochloremia may contribute to cardiovascular collapse, emphasizing the urgent need for prompt correction of electrolyte imbalances to restore cardiac stability. Monitoring chloride levels becomes critical in preventing and managing potentially life-threatening arrhythmias in critically ill patients [23].

Microcirculation disturbances: Both hyperchloremia and hypochloremia have been linked to disturbances in microcirculation, impacting tissue perfusion at the cellular level. Hyperchloremia-induced decreases in cardiac output can lead to compromised microcirculation, affecting oxygen and nutrient delivery to tissues. Similarly, hypochloremia-associated arrhythmias can disrupt the coordinated flow of blood through microvessels. These disturbances in microcirculation contribute to organ dysfunction and compromise overall patient outcomes. Recognizing the implications of chloride imbalances on microcirculation is essential for clinicians managing critically ill patients, as it informs interventions to optimize tissue perfusion and mitigate organ damage [27].

Strategies for chloride management

Fluid Resuscitation Protocols

Goal-directed therapy: Implementing goal-directed fluid therapy is fundamental in managing critically ill patients. This strategy involves tailoring fluid administration to specific clinical endpoints based on individual patient needs. Factors such as hemodynamic status, urine output, and perfusion markers are carefully monitored to guide fluid resuscitation. By setting and adjusting therapeutic goals in response to real-time patient parameters, clinicians can optimize fluid balance, mitigate the risk of overhydration, and minimize the potential for electrolyte imbalances, including hyperchloremia. Goal-directed therapy enhances the precision of fluid management, promoting individualized care in complex clinical scenarios [28].

Limiting normal saline use: The association between hyperchloremia and normal saline administration has prompted clinicians to reconsider fluid choices. Limiting normal saline, a chloride-rich solution, is a proactive measure to reduce the risk of hyperchloremic acidosis and other electrolyte imbalances. Balanced crystalloids, which contain a more physiologically balanced composition of electrolytes, are increasingly favored as alternatives to normal saline. By selecting fluids that align more closely with the body's natural electrolyte balance, clinicians aim to minimize the adverse effects associated with hyperchloremia, providing a safer approach to fluid resuscitation in critically ill patients [29].

Dynamic versus static indicators: Dynamic indicators, such as stroke volume variation or pulse pressure variation, offer a more sophisticated approach to fluid resuscitation than static parameters alone. Dynamic indicators provide real-time insights into changes in intravascular volume and cardiac preload, allowing for more precise adjustments in fluid administration. This approach reduces the risk of overhydration and hyperchloremia, mainly when static parameters may not accurately reflect the patient's fluid responsiveness. Incorporating dynamic indicators into fluid management strategies enhances the clinician's ability to optimize intravascular volume, tailor interventions to individual patient needs, and minimize the potential for electrolyte imbalances [30].

Use of Crystalloids Versus Colloids

Crystalloids as first-line options: Crystalloid solutions encompassing normal saline and balanced crystalloids are frequently employed as first-line options for fluid resuscitation in various clinical settings. These solutions have several advantages: ready availability, cost-effectiveness, and good patient tolerability. Normal saline, containing 0.9% sodium chloride, is commonly used in emergencies and critical care due to its simplicity and immediate availability. Balanced crystalloids, which mimic plasma electrolyte composition more closely, are increasingly favored to mitigate the risk of hyperchloremia associated with normal saline. The versatility and familiarity of crystalloid solutions make them essential components in fluid resuscitation protocols, providing a foundation for initial interventions in critically ill patients [31].

Colloids in specific situations: Colloids such as albumin and synthetic solutions such as hydroxyethyl starch may be considered in specific situations and patient populations. Colloids are more expensive than crystalloids. They are better than crystalloids at reducing death, need for blood transfusion, or need for renal replacement therapy (filtering the blood, with or without dialysis machines, if kidneys fail) when given to critically ill people who need fluid replacement. Colloids can expand intravascular volume more effectively than crystalloids due to their larger molecular size. They may be instrumental in cases of severe hypovolemia or patients at risk of fluid overload, as they require smaller volumes for similar volume-expanding effects. However, the choice between crystalloids and colloids remains a subject of ongoing debate. It is highly dependent on individual patient characteristics, the clinical context, and considerations of safety and cost-effectiveness. The potential risks associated with colloids, such as coagulopathy and renal dysfunction, must be carefully weighed against their potential benefits in specific situations [32].

Role of Balanced Solutions

Reducing hyperchloremic acidosis: The risk of hyperchloremic acidosis associated with normal saline has led to increased consideration of balanced crystalloid solutions. These solutions, such as lactated Ringer's or Plasma-Lyte, aim to mimic the electrolyte composition of plasma more closely. By providing a more balanced ratio of electrolytes, including lower chloride concentrations, they offer an alternative to normal saline with the potential to reduce the risk of hyperchloremic acidosis. In scenarios where careful chloride management is crucial, such as in patients prone to renal dysfunction or those with pre-existing acid-base imbalances, clinicians may opt for balanced crystalloid solutions to minimize the adverse effects associated with hyperchloremia [33].

Electrolyte composition consideration: The consideration of electrolyte composition is a pivotal aspect of tailoring fluid regimens to individual patient needs. Balanced crystalloid solutions with lower chloride content are vital to maintaining electrolyte balance and acid-base homeostasis. While normal saline may have a chloride concentration of 154 mmol/L, balanced solutions offer a more nuanced approach. For example, lactated Ringer's solution contains chloride at approximately 109 mmol/L. Clinicians should carefully assess patient characteristics, underlying conditions, and the specific electrolyte requirements of each case when selecting fluid options. By considering the solutions' electrolyte composition, healthcare providers can optimize fluid management strategies, minimizing the risk of electrolyte imbalances and associated complications [34].

Individualized Patient Approaches

Assessing patient risk factors: Recognizing patient-specific risk factors for chloride imbalances is foundational in preventing adverse outcomes. Risk factors may include pre-existing renal dysfunction, electrolyte disturbances, or conditions predisposing individuals to fluid imbalances. A thorough assessment of these factors allows clinicians to tailor chloride management strategies to individual patient needs. For example, minimizing hyperchloremic solutions may be prioritized in patients with impaired renal function to mitigate the risk of further renal complications. A nuanced understanding of patient risk factors informs proactive interventions to prevent chloride imbalances and associated complications [35].

Continuous monitoring: Continuous monitoring of chloride levels, especially in high-risk patients, is essential for early detection and intervention. This monitoring can be achieved through regular laboratory testing or point-of-care testing devices for real-time results. High-risk patients, such as those with renal dysfunction or receiving large volumes of fluids, may benefit from more frequent monitoring to ensure timely identification of deviations from normal chloride levels. Continuous surveillance enables healthcare providers to proactively address imbalances, preventing the progression to more severe electrolyte disturbances and optimizing patient outcomes [36].

Adapting to changing conditions: Critical care patients often experience dynamic changes in their clinical status, necessitating fluid and chloride management strategies adaptable to evolving conditions. Regular reassessment and adjustment of fluid regimens based on patient response are critical components of effective management. As the patient's clinical status changes, such as in response to interventions or resolving underlying conditions, healthcare providers should be prepared to modify fluid and chloride administration accordingly. This adaptability ensures that interventions remain aligned with the patient's evolving needs, reducing the risk of under- and overcorrection of chloride imbalances [37].

Clinical trials and evidence-based practices

Overview of Relevant Clinical Studies

Managing chloride levels is critical to providing care to patients, particularly in critical care settings such as intensive care units (ICUs). Dyschloremia, which refers to an imbalance in chloride levels, can manifest due to various factors both before and during a patient's admission to the ICU [38]. Both hypo- and hyperchloremia, representing lower and higher than normal chloride levels, respectively, have been linked to unfavorable clinical outcomes, including increased mortality and the occurrence of acute kidney injury (AKI) [38,39]. Recognizing the significance of chloride concentration in resuscitation fluids used in the ICU, researchers have dedicated attention to investigating this aspect of patient care. The field of research has specifically explored the comparative effectiveness and safety of balanced crystalloid solutions versus saline for fluid therapy in critically ill adults [38-40].

Studies have been conducted across various patient populations, examining outcomes such as mortality, AKI, nutritional considerations, and factors such as coagulopathy and transfusion requirements [38]. Despite these research efforts, uncertainties remain regarding the optimal choice between balanced crystalloid solutions and saline for fluid resuscitation in critically ill adults. This underscores the complexity of chloride management in critical care, necessitating ongoing research and a nuanced approach to address the diverse needs of patients in the ICU.

Key Findings and Controversies

Clinical research has revealed key insights into chloride management in critical care, emphasizing three major findings. Firstly, dyschloremia, encompassing both hypo- and hyperchloremia, has been consistently linked to unfavorable clinical outcomes. Notably, these outcomes include increased mortality rates and a higher incidence of acute kidney injury (AKI) [38,39]. The chloride concentration in resuscitation fluids administered within the intensive care unit (ICU) has emerged as a critical factor influencing patient outcomes. This recognition underscores the importance of carefully considering the composition of fluids used in fluid therapy for critically ill adults. The determination of the optimal choice between balanced crystalloid solutions and saline remains uncertain despite extensive research efforts [38-40]. A contentious issue surrounds using chloride-rich solutions, particularly in the context of extensive volume fluid resuscitation in the ICU. Some recommendations propose that fluids with lower chloride content, such as lactated Ringer's solution, may be equally effective, offering the additional advantage of avoiding hyperchloremia, which has been associated with adverse outcomes [39]. These findings collectively highlight the complexity of chloride management in critical care. Continued research and a nuanced, individualized approach to fluid therapy are essential, considering the specific clinical context and the potential impact of chloride levels on patient outcomes.

Gaps in Current Knowledge

Despite the wealth of existing research, knowledge gaps persist in chloride management for critically ill patients. The precise mechanisms and optimal strategies for managing dyschloremia are yet to be fully comprehended. In particular, there is an ongoing need for a deeper understanding of the intricacies surrounding dyschloremia and how best to manage it effectively. The comparative efficacy and safety of various fluid therapy options, such as balanced crystalloid solutions and saline, remain subjects of uncertainty that necessitate further exploration [40]. This underscores the complexity of fluid management decisions in critical care settings and highlights the ongoing quest for clarity in choosing the most appropriate and beneficial options for patient care. Moreover, the long-term effects of dyschloremia on patient outcomes represent another area requiring extensive investigation. Understanding how dyschloremia may influence patients over an extended period is crucial for developing comprehensive and effective care strategies. Additionally, there is a need to explore whether improvements in patient care can be achieved through more targeted and nuanced chloride management approaches [41].

Practical considerations in chloride management

Monitoring and Surveillance

Regular laboratory monitoring: Implementing a routine schedule for monitoring chloride levels is fundamental to chloride management. A regular schedule for blood tests measuring serum chloride concentrations may be established depending on the clinical context and patient characteristics. Daily or more frequent measurements may be warranted in critical care settings, where electrolyte imbalances can have swift and significant consequences. This approach allows for the timely identification of deviations from normal chloride levels, enabling healthcare providers to intervene proactively and prevent adverse outcomes, especially in high-risk patients or those undergoing significant interventions [42].

Point-of-care testing: Point-of-care testing devices for immediate assessment of chloride levels at the bedside are a valuable strategy, particularly in emergencies or when rapid decision-making is crucial. Point-of-care testing provides real-time results, allowing clinicians to make prompt interventions based on the patient's electrolyte status. This approach is especially beneficial in critical care scenarios where time is of the essence, offering a quick and efficient means of assessing chloride levels without the delay associated with traditional laboratory processing. Point-of-care testing enhances the agility of healthcare teams in responding to acute changes in chloride balance [43].

Integration into electronic health records (EHRs): Integrating chloride monitoring data into electronic health records (EHRs) is a strategic measure that enhances accessibility and promotes a comprehensive view of the patient's electrolyte status over time. This integration facilitates trend analysis, allowing healthcare providers to track changes in chloride levels, identify patterns, and make informed decisions about patient management. It also supports communication among healthcare team members by providing a centralized and readily accessible source of information. Integrating chloride monitoring into EHRs contributes to a cohesive approach to patient care, ensuring that relevant data is available to all involved providers for comprehensive decision-making [44].

Challenges in Implementation

Fluid overload concerns: Striking a delicate balance between maintaining adequate fluid status and avoiding fluid overload is a common challenge in critical care. Clinicians must be vigilant to prevent iatrogenic hyperchloremia, primarily by administering chloride-rich fluids. The risk of fluid overload is particularly pertinent in patients with compromised cardiac or renal function. Careful consideration of individual patient factors, ongoing assessment of fluid responsiveness, and the judicious use of fluids, including selecting appropriate solutions, are essential to managing this delicate balance [45].

Establishing consistent protocols: Developing and implementing consistent chloride management protocols across diverse healthcare settings and among different care providers can be a challenging endeavor. Standardized guidelines, supported by evidence-based practices, can help establish uniformity in chloride management. Educational initiatives targeting healthcare professionals in fluid resuscitation and electrolyte management contribute to adopting standardized protocols. Implementing consistent protocols ensures that best practices are applied across various clinical scenarios, promoting quality and safety in patient care [46].

Resistance to change: The transition from traditional practices, such as the routine use of normal saline, to newer strategies involving balanced solutions may encounter resistance. Clinicians may be accustomed to established routines, and changing practices requires overcoming ingrained habits. Fostering a culture of evidence-based practice, backed by education and clear communication of the benefits of alternative strategies, is crucial for overcoming resistance to change. Providing clinicians with the rationale behind new protocols and demonstrating their impact on patient outcomes help build confidence in adopting updated approaches to chloride management [47].

Multidisciplinary Approach

Collaboration among healthcare providers: A multidisciplinary approach involving physicians, nurses, pharmacists, and other allied health professionals is crucial for holistic chloride management. Regular communication and collaborative decision-making ensure a comprehensive understanding of the patient's condition and contribute to developing well-rounded care plans. Each healthcare provider brings a unique perspective, and collaboration fosters a synergistic approach to chloride management, enhancing the overall quality of patient care [48].

Education and training: Ongoing education and training initiatives are essential for healthcare teams to stay informed about the importance of chloride balance, the risks associated with imbalances, and the latest evidence-based strategies for chloride management. Continuous learning empowers healthcare professionals to adapt to evolving practices and integrate new knowledge into clinical decision-making. By investing in education and training, healthcare institutions promote a culture of continuous improvement, ensuring that providers are equipped with the knowledge and skills needed for effective chloride management [49].

Incorporating nutrition and pharmacy services: Nutritionists and pharmacists play pivotal roles in chloride management, particularly in patients receiving enteral or parenteral nutrition. These professionals contribute to comprehensive patient care by calculating chloride intake from nutrition sources, adjusting nutrition regimens based on patient needs, and selecting medications with consideration for their chloride content. Incorporating nutrition and pharmacy services into chloride management strategies ensures a well-rounded approach that addresses both dietary and pharmacological sources of chloride [50].

Shared decision-making with patients: Involving patients in discussions about their care, including the rationale for specific chloride management strategies, promotes shared decision-making. Transparent communication with patients regarding the risks and benefits of different interventions fosters patient engagement and compliance. When patients understand the importance of chloride balance and the reasons behind certain management decisions, they are more likely to actively participate in their care and adhere to recommended treatment plans [51].

Future directions and research needs

Emerging Therapies and Interventions

Selective chloride modulators: Investigating the development of selective chloride modulators or agents specifically targeting chloride channels represents a potential avenue for fine-tuned chloride regulation. By focusing on selective modulation, researchers aim to intervene at the molecular level, allowing for more precise control over chloride levels. This approach holds promise in mitigating the risk of adverse effects associated with broad-spectrum interventions, potentially minimizing the impact on other electrolytes and physiological processes. Selective chloride modulators could offer a targeted and tailored approach to chloride management in critically ill patients [52].

Precision fluid resuscitation: Advancements in technology and patient monitoring systems may enable more precise, individualized fluid resuscitation strategies. Continuous real-time monitoring of the patient's fluid status, electrolyte levels, and hemodynamic parameters could provide a dynamic and personalized approach to chloride management. This precision fluid resuscitation, guided by real-time data, can potentially optimize chloride balance while minimizing the risk of imbalances. Tailoring interventions to individual patient responses and needs may lead to more effective and safer fluid management in critical care settings [53].

Innovations in intravenous fluids: Ongoing research into novel intravenous fluids with optimized electrolyte compositions, including lower chloride content, promises to improve chloride management in critically ill patients. Innovations in fluid formulations that better mimic physiological conditions may contribute to reduced risks of electrolyte imbalances and associated complications. These advancements could lead to intravenous fluids that are more tailored to the specific needs of critically ill patients, optimizing fluid resuscitation and overall patient outcomes [34].

Areas for Further Investigation

Long-term impact of chloride management: Understanding the long-term consequences of chloride management strategies is crucial for comprehensive patient care. Research efforts should explore the impact of different interventions on patient outcomes beyond the acute phase. This includes investigating potential effects on long-term renal function, cardiovascular health, and overall mortality. Longitudinal studies can provide valuable insights into the sustained impact of chloride management practices, guiding clinicians in making informed decisions that consider immediate outcomes and the patient's long-term well-being [54].

Subpopulations and vulnerable patient groups: Investigating chloride management in specific patient populations, such as those with pre-existing renal dysfunction, the elderly, or pediatric patients, is essential for tailoring interventions to individual needs. Vulnerable patient groups may respond differently to chloride management strategies, and understanding these nuances is crucial for optimizing clinical care. Research should address the unique considerations and potential risks associated with chloride imbalances in different subpopulations, facilitating the development of personalized and targeted approaches [55].

Economic and resource utilization impact: Assessing the economic implications and resource utilization associated with various chloride management strategies is vital for healthcare systems. Comparative effectiveness research can guide decision-makers in identifying cost-effective and efficient approaches to chloride management. Understanding the economic impact of different interventions, including the costs associated with complications and prolonged hospital stays, can inform healthcare policies and resource allocation, ultimately contributing to the sustainability and efficiency of healthcare delivery [56].

Potential Impacts on Critical Care Guidelines

Incorporation of balanced solutions: As the evidence supporting the use of balanced crystalloids continues accumulating, future updates to critical care guidelines may prioritize these solutions over traditional chloride-rich options. Guidelines could evolve to emphasize the preference for balanced crystalloids in various clinical scenarios, considering their potential benefits in mitigating the risk of hyperchloremia. Specific recommendations based on patient characteristics, such as renal function and comorbidities, may be provided to guide clinicians in selecting the most appropriate fluid resuscitation options [33].

Integration of individualized approaches: Future critical care guidelines may increasingly emphasize the importance of individualized chloride management based on patient-specific factors. This shift reflects a recognition of the heterogeneity of patient populations and the need for a more nuanced, personalized approach to patient care. Guidelines may provide frameworks for tailoring chloride management strategies based on age, underlying health conditions, and the nature of critical illness. This individualized approach could optimize patient outcomes by considering each patient's unique needs and risks [37].

Continuous monitoring recommendations: Recognizing the value of continuous monitoring, future critical care guidelines may recommend incorporating real-time assessments of chloride levels, particularly in high-risk patients. Continuous monitoring allows dynamic fluid and chloride management adjustments based on evolving patient conditions. Guidelines may provide recommendations for using point-of-care testing devices and other monitoring technologies to enable healthcare providers to identify and promptly address deviations from normal chloride levels. This proactive approach could help prevent the development of severe electrolyte imbalances and associated complications [57].

## Conclusions

In conclusion, chloride management in critically ill patients represents a dynamic and multifaceted aspect of clinical care. This comprehensive review has underscored the significance of chloride in maintaining physiological homeostasis, explored the pathophysiology of imbalances, detailed strategies for clinical assessment, and delved into the consequences of dysregulated levels. The synthesis of critical points emphasizes the importance of individualized patient care, thoughtful selection of intravenous fluids, and continuous monitoring practices. As clinicians navigate this complex landscape, future directions and research needs open avenues for precision therapies, personalized approaches, and potential impacts on critical care guidelines. The implications for clinical practice stress the need for adaptability, evidence-based decision-making, and a commitment to advancing patient outcomes. In closing, the ever-evolving field of chloride management requires a collaborative and informed approach, where healthcare providers remain vigilant, continuously educate themselves on emerging developments, and uphold a dedication to enhancing the well-being of critically ill individuals.
